# Bioaccumulation of CdSe Quantum Dots Show Biochemical and Oxidative Damage in Wistar Rats

**DOI:** 10.1155/2023/7707452

**Published:** 2023-04-06

**Authors:** Kishan Das, Ramovatar Meena, Usha Singh Gaharwar, Eepsita Priyadarshini, Kamla Rawat, R. Paulraj, Yugal Kishore Mohanta, Muthupandian Saravanan, Himadri B. Bohidar

**Affiliations:** ^1^Shaheed Rajguru College of Applied Sciences for Women, University of Delhi, New Delhi, India; ^2^School of Physical Sciences, Jawaharlal Nehru University, New Delhi, India; ^3^School of Environment Sciences, Jawaharlal Nehru University, New Delhi, India; ^4^Swami Shraddhanand College, University of Delhi, Delhi, India; ^5^Department of Chemistry, School of Life and Chemical Sciences, Jamia Hamdard, New Delhi, India; ^6^Department of Applied Biology, School of Biological Sciences, University of Science and Technology Meghalaya (USTM), 9th Mile, Baridua-793101, Ri-Bhoi Dist., Meghalaya, India; ^7^Department of Medical Microbiology and Immunology, Division of Biomedical Sciences, School of Medicine, College of Health Sciences, Mekelle University, Tigray, Ethiopia; ^8^AMR and Nanotherapeutics Laboratory, Department of Pharmacology, Saveetha Dental College, Saveetha Institute of Medical and Technical Sciences (SIMATS), Chennai, 600 077 Chennai, India

## Abstract

Cadmium selenium quantum dots (CdSe QDs) with modified surfaces exhibit superior dispersion stability and high fluorescence yield, making them desirable biological probes. The knowledge of cellular and biochemical toxicity has been lacking, and there is little information on the correlation between *in vitro* and *in vivo* data. The current study was carried out to assess the toxicity of CdSe QDs after intravenous injection in Wistar male rats (230 g). The rats were given a single dose of QDs of 10, 20, 40, and 80 mg/kg and were kept for 30 days. Following that, various biochemical assays, hematological parameters, and bioaccumulation studies were carried out. Functional as well as clinically significant changes were observed. There was a significant increase in WBC while the RBC decreased. This suggested that CdSe quantum dots had inflammatory effects on the treated rats. The various biochemical assays clearly showed that high dose induced hepatic injury. At a dose of 80 mg/kg, bioaccumulation studies revealed that the spleen (120 g/g), liver (78 g/g), and lungs (38 g/g) accumulated the most. In treated Wistar rats, the bioretention profile of QDs was in the following order: the spleen, liver, kidney, lungs, heart, brain, and testis. The accumulation of these QDs induced the generation of intracellular reactive oxygen species, resulting in an alteration in antioxidant activity. It is concluded that these QDs caused oxidative stress, which harmed cellular functions and, under certain conditions, caused partial brain, kidney, spleen, and liver dysfunction. This is one of the most comprehensive *in vivo* studies on the nanotoxicity of CdSe quantum dots.

## 1. Introduction

Nanoparticles have demonstrated wide applications because of their small size and large surface-to-volume ratio attributes [1]. The last decade has witnessed a revolution in the synthesis, engineering, and application of these nanoscale materials in domains ranging from cosmetics, food and packaging, ceramics, drug delivery, bioimaging, and cancer treatment [2, 3]. Currently, the most commercially important nanomaterials include carbon-based nanoparticles, graphene nanomaterials, fullerenes, and quantum dots. Among these nanomaterials, CdSe quantum dots and carbon-based nanotubes, because of their stability and high fluorescence, have shown promising biomedical applications. Therefore, there has been an exponential rise in the synthesis and surface engineering of these nanomaterials to optimize their use in the form of biosensors, tumor detectors, or bioimaging probes [3–6]. However, these applications involve direct interaction with human cells and tissues, thus raising uncertainties about the health effects and associated risks. The interaction of small nanoparticles like quantum dots can have a severe toxic effect because they can induce the generation of reactive oxygen species (ROS), deregulate mitochondrial functioning, and severely affect cellular metabolism [7–9].

The small size of CdSe QDs assists them to easily cross the cellular membrane and internalizing in the cytosolic space, specifically in lysosomes [10–13]. The acidic pH of around 4.5 in the lysosomes facilitates the degradation of QDs, thereby releasing free Cd^2+^ ions into the surrounding medium.^10^ These Cd^2+^ ions in turn stimulate the generation of ROS that in turn affects cellular metabolism and may initiate apoptosis [10, 11, 14]. Additionally, reports also suggest that QDs can interact specifically with biomolecules, thereby, resulting in DNA damage, protein dysfunction which in some cases may lead to neurotoxicity by interacting with hematological factors [15–17]. The ingestion and accumulation of these particles in vital organs like the spleen, kidney, liver, and lungs can result in changes at the cellular, subcellular, and molecular levels [18, 19].

Due to their consistent fluorescence characteristics, cadmium-based QDs have attracted a lot of interest in the past due to their potential in both biomedical and clinical applications. Due to the significant impact that size, shape, and composition have on QD properties and subsequent toxicity behavior, although studies have correlated the toxicity of such QDs concerning size and surface coating, the determination of the influence of these QDs on the biochemical and genotoxicity parameters *in vivo* remains somewhat poorly understood. Reports suggest that coating QDs with capping agents such as BSA, PEG, or glutathione makes them less toxic and drastically reduces ROS generation [14, 20, 21]. Nonetheless, the smaller size of QDs allows their translocation to bloodstream allowing uptake and accumulation in various organs [22, 23]. Previous studies reported that Cd^2+^ ions from Cd-based nanoparticles can cause hepatic injury by specifically binding with sulfhydryl groups of mitochondrial proteins [24] and that Cd^2+^ ions in the concentration range of 100–400 nmol/mL are potentially toxic and lead to cell death [25]. Previous studies have shown that CdSe QDs are toxic in vitro because they cannot mimic the real *in vivo* environment and underlying reactions. The exposure to human tissues is also increased by the use of CdSe QDs in imaging, diagnosis, and drug delivery. When these nanoparticles are exposed to an *in vivo* system, it is crucial to determine their *in vivo* toxicity, biodistribution, and clearance profiles. Additionally, the properties and mechanisms of toxicity of QDs can vary significantly *in vivo* and *in vitro* systems. While some studies reported a direct correlation between cadmium accumulations concerning time after intravenous injection [26], other studies signified nontoxicity of CdSe/ZnS QDs even after short- and long-term exposure of 7 and 80 days, respectively [27].

Hence, it is imperative to determine the toxicity of CdSe QDs before ascertaining their biological applications. We have used CdSe quantum dot-Wistar rat (8–10 weeks old) as the model system to investigate the bioaccumulation-induced biochemical toxicity after intravenous administration. Considering the potential use of quantum dots in pharmaceutical formulations, the aforesaid study is of relevance. Further, the depth and breadth of the present *in vivo* study impart sufficient novelty to the work reported.

## 2. Results

### 2.1. QD Characterizations

The prepared QDs were characterized by UV-visible absorption, fluorescence spectroscopy, X-ray diffraction, transmission electron microscope, and EDAX techniques to determine their morphology, size, and crystallinity parameters. The details are provided in our earlier work [28]. In brief, these were nanocrystalline spherical particles of 1 : 1 (atomic percentage) elemental composition of Cd : Se, and mean size of 2.5 nm, possessing a zeta potential of −60 mV. The absorption and emission peaks were 474 and 617 nm, respectively.

### 2.2. Body Weight and Organ Coefficient

**Figure 1:**
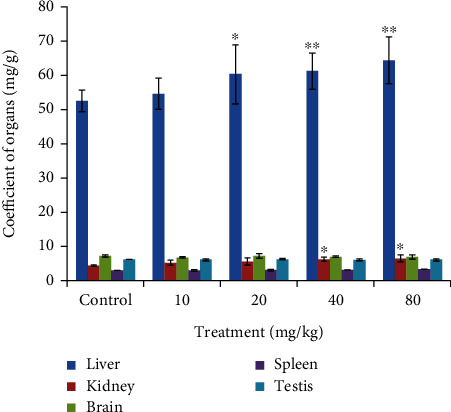
Organ coefficient (the liver, kidney, testis, spleen, and brain) of control and treated set of Wistar rats. Organ coefficient is the ratio of weight of organs (mg) to weight of animals (g). ^∗^Statistically significant results at *p* < 0.05.

As per the study, increasing doses of CdSe QDs were administered to the rats and assays were performed at regular intervals. Administration of CdSe QDs did not produce significant changes in the feed and water intake of Wistar rats. No unusual response, behavioral changes, or death of the animal were observed throughout the experiment period. No weight loss was observed in the treated group of animals.  Figure 1 shows the relative organ-to-body weight ratio of the animals treated with varying doses of QDs. No significant changes in the organ coefficient of the brain, spleen, testis, and kidney were observed in treated groups, except for the treated liver. The organ coefficient for the liver was found to increase with a higher dose of QDs injected.

**Figure 2:**
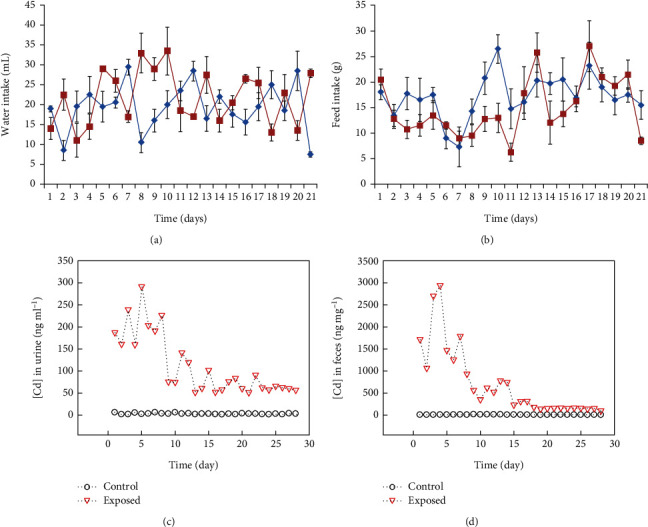
Metabolic response of the Wistar rats (a) depicts water intake and (b) depicts food intake, with block lines representing the control and red line treated groups, respectively, graph showing the concentration of Cd in (c) urine and (d) feces in the control and exposed groups of rats.

No major changes in the food and water uptake were found in the treated group of rats as can be observed in Figures 2(a) and 2(b).

### 2.3. Excretion of CdSe QDs through Urine and Feces

CdSe QD concentration was also estimated in urine and feces samples of the rats by using AAS analysis. For this, all the control and treated animals were kept in metabolic cages and the samples were collected daily. Further, AAS analysis of Cd concentration in the collected urine and feces was performed. As can be seen from Figures 2(c) and 2(d), higher amount of Cd was excreted in urine and feces in the first week of treatment. Additionally, we noticed significant elimination of Cd in fecal matter when compared to the urine in the initial week of treatment along with the intravenous administration. Moreover, it can be suggested that the feces were the main route of elimination of Cd [29]. With an increasing dose of QD administered, increasing amount of Cd was found to be eliminated in urine and feces.

### 2.4. Bioaccumulation of CdSe QDs

**Figure 3:**
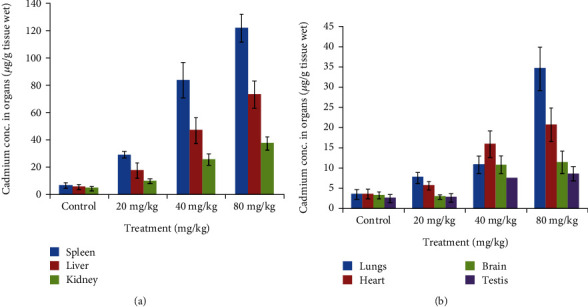
Graph depicting bioaccumulation of cadmium in different organs, with the spleen showing the maximum accumulation and testis the least.

**Figure 4:**
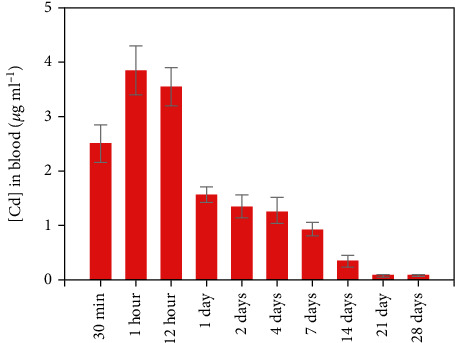
Graph showing the concentration of Cd in blood of Wistar rats administered with CdSe QDs.

Bioaccumulation of nanoparticles occurs when the rate of uptake is greater than the rate of clearance. However, the route of administration, its biopersistence, and nanoparticle properties also affect bioaccumulation [30]. Cd concentration in different organs was estimated using AAS analysis. Results signified that Cd was accumulated in all the studied organs. As can be seen from Figure 3, the spleen and liver showed the highest Cd concentration across the dose administered. Cd concentration is presented as *μ*g/g of tissue weight. The order of bioaccumulation of Cd was as spleen>liver>kidney>lungs>heart>brain>testis. At maximum, for CdSe dose (80 mg/kg), the spleen showed the highest bioaccumulation (120 *μ*g/g) and the testis, the least (10 *μ*g/g). Bioaccumulation has also been observed in lower doses in different organs. However, those changes were not statistically significant to report. Additionally, we estimated the concentration of Cd in the blood of the treated groups. As can be observed in Figure 4, the concentration of Cd was high during the first day of administrating QDs. Approximately, 3.8 *μ*g/mL Cd was estimated after 12 hours of treatment with QDs. The concentration of Cd was found to decrease thereafter and was minimal after 21 days of treatment.

### 2.5. Hematological Changes

**Figure 5:**
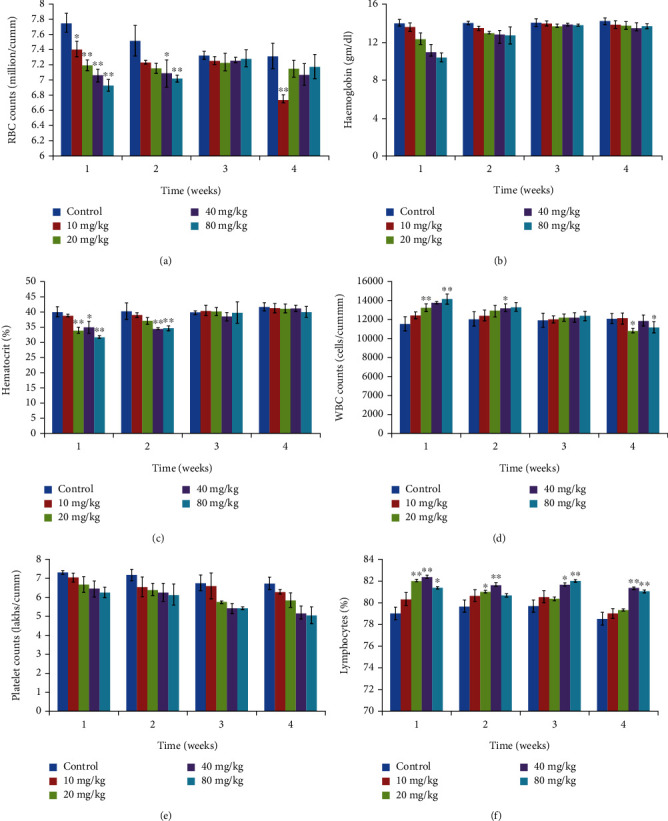
Hematological results from animals treated with varying dose of CdSe QDs. (a) Red blood cells (millions/cumm). (b) Hemoglobin (g/dL). (c) Hematocrit (%). (d) White blood cells (1000 cells/cumm). (e) Platelet counts (lakhs/cumm). (f) Lymphocytes (%). The results show the mean and standard deviation of six independent readings.

The different hematological parameters (RBC, WBC, hemoglobin, hematocrit, platelet, and lymphocyte) were analyzed at weekly intervals, and the results have been shown in Figures 5(a)–5(f). In the first week of treatment, significant changes in RBC count, hemoglobin, and hematocrit % were noticed as compared to the control group. With an increasing dose of CdSe administered, a considerable decrease in these parameters was found. Yet, in later weeks (3^rd^ and 4^th^), the count was almost the same as that of the control group. This signifies that administration of CdSe induced immunogenic changes in Wistar rats, but with time body adapted to such changes. Similarly, we observed a decrease in WBC count with increasing dose in the first week of administration, and not much variation from the control group was found thereafter. RBC count decreased from around 7.7 million/cumm (control) to 6.9 million/cumm, while WBC increased from 11,500 million/cumm (control) to around 14,000 million/cumm (80 mg/kg CdSe). Thus, dose-dependent decrease in RBC along with a concomitant increase in WBC count suggested the toxic effects of these QDs on the rats.

### 2.6. Biochemical Assays for Organ Functioning

**Figure 6:**
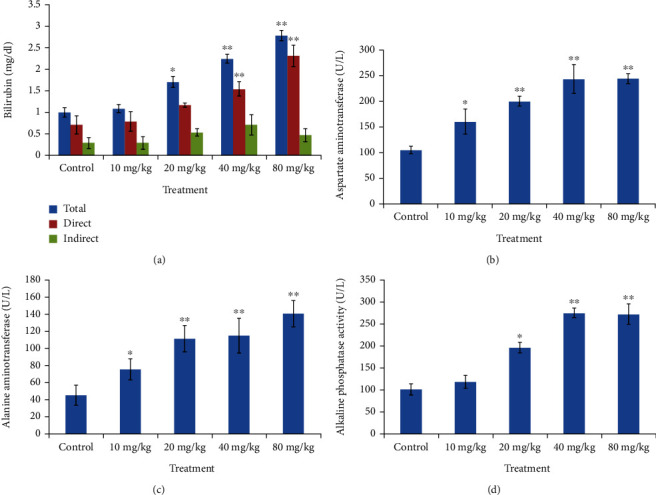
Graph depicting changes in liver function markers from Wistar rats treated with varying dose of CdSe QDs. (a) Bilirubin (total, direct, and indirect). (b) Asparate aminotransferase (AST). (c) Alanine aminotransferase (ALT). (d) Alkaline phosphatase (ALP). ^∗^Statistically significant at (*p* < 0.05), ^∗∗^very significant (*p* < 0.001).

Analysis of various biomarkers that serve as markers for kidney and liver functioning was conducted. We analyzed the levels of bilirubin (total, direct, and indirect), aspirate aminotransferase (AST), alanine aminotransferase (ALT), and alkaline phosphatase (ALP), which give indications of liver damage. In all the cases, significant changes were observed in the treated rat group. The level of ALT, AST, and ALP increased significantly in the experimental group. While significant changes (*p* < 0.05) were found at 10 mg/kg CdSe concentration, the changes were more prominent (p<0.001) at 20, 40, and 80 mg/kg CdSe concentration. Likewise, bilirubin concentration that provides a direct indication of liver functioning also showed a very significant (*p* < 0.001) increase in 40 and 80 mg/kg of CdSe QDs-treated groups. The results are presented in Figure 6.

**Figure 7:**
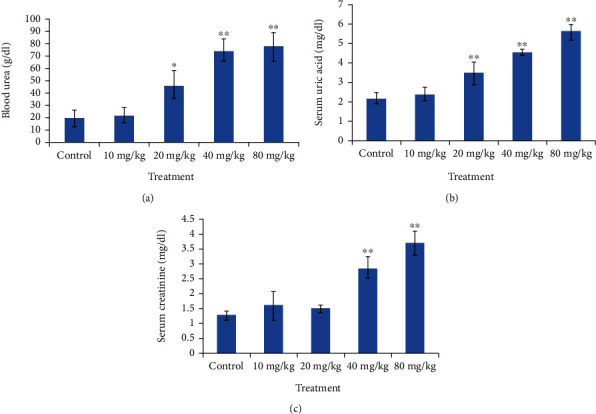
Graph depicting dose dependent changes in kidney function markers in the treated Wistar rates. (a) Blood urea. (b) Uric acid. (c) Creatinine. ^∗^Significant (*p* < 0.05) and ^∗∗^very significant changes (*p* < 0.001).

Analysis of urea, serum uric acid, and creatinine metabolites was done to determine any indication of kidney-related damage (Figure 7). At high CdSe QD concentration, a significant increase in the level of these kidney metabolites was observed compared to that of the control. Dose-dependent increase in urea and uric acid concentration was also noticed.

### 2.7. Effect of CdSe QDs on Antioxidative System

**Figure 8:**
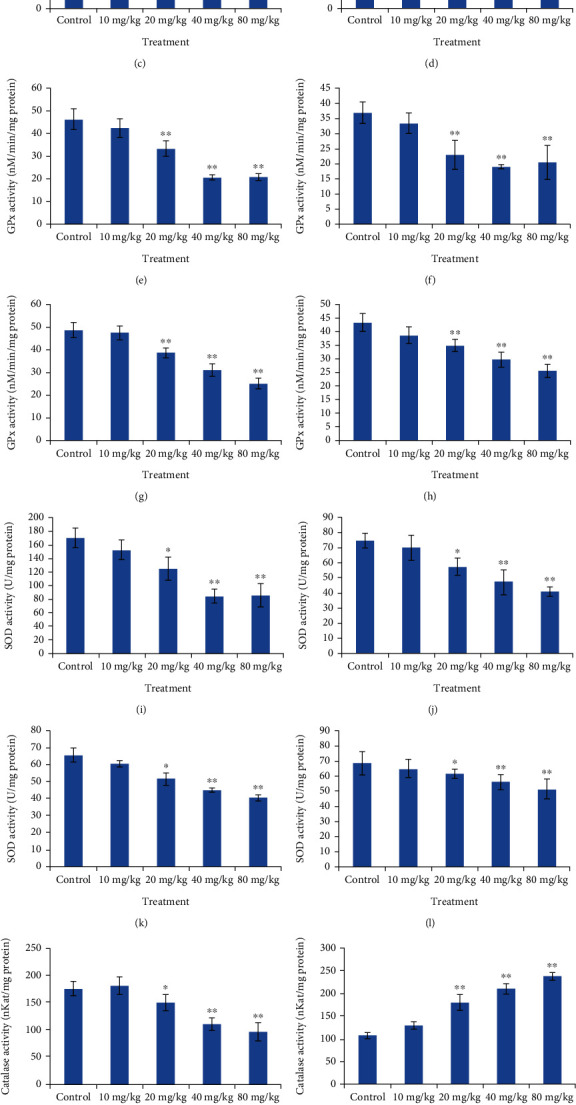
Graph depicting comparison of antioxidative enzyme (the liver, kidney, and brain, respectively) in the control and CdSe QDs-treated Wistar rats. Lipid peroxidise activity in the (a) liver, (b) kidney, (c) brain, and (d) spleen. GPx activity in the (e) liver, (f) kidney, (g) brain, and (h) spleen. SOD activity in the (i) liver, (j) kidney, (k) brain, and (l) spleen. Catalase activity in the (m) liver, (n) kidney, (o) brain, and (p) spleen.

Nanoparticles are known to exert toxic effects on cells by the generation of free radicals, which then induce oxidative stress [31–33]. As the experimental results suggested that the QDs induce damage at a higher concentration, we hypothesized that ROS-mediated oxidative stress can be the basic mechanism behind the toxicity. Hence, we studied the generation of ROS by QDs by estimation of the liver, brain, spleen, and kidney antioxidative enzymes that included catalase, SOD, lipid, and malondialdehyde. The generation of malondialdehyde is used as an indication of lipid peroxidation. The level of lipid peroxidises significantly increased with higher doses (40 and 80 mg/kg) of QD administered to the rats, thus signifying that the QDs induced lipid peroxidation (Figures 8(a)–8(d)). Graph (Figures 8(e)–8(h)) shows the effect of QDs on glutathione peroxidise activity in the brain, kidney, liver, and spleen. Except for lower dose (10 mg/kg), 20, 40, and 80 mg/kg CdSe QDs-treated groups showed a significant decrease in GPx level as compared to the control. Similarly, a reduction in the SOD activity was found when compared to that of the control in the liver, kidney, spleen, and brain. A dose-dependent reduction in SOD level was observed with prominent changes observed in 40 mg/kg- and 80 mg/kg-treated groups (Figures 8(i)–8(l)). The effect of QDs on catalase activity is presented in Figures 8(m)–8(p). A significant reduction (*p* < 0.05) in the liver, spleen, and brain catalase activity was observed compared to the control set. At a higher dose of CdSe, the reduction was very significant (*p* < 0.001) indicating the capability of CdSe QDs to inhibit the catalase activity in a dose-dependent manner. However, we noticed an enhancement in kidney catalase activity compared to the control set. Thus, the QDs induced oxidative stress which was evident from the marked reduction in GPx, catalase, SOD levels, and enhanced lipid peroxidation. It is well known that the generation of ROS (superoxide, hydroxyl radical, and hydrogen peroxide) instigate a series of cellular responses such as inflammation, DNA damage, and cellular apoptosis [34].

## 3. Discussion

QD has become a promising tool for cell tracking and diagnostic purpose both *in vitro* and *in vivo* [35]; however, QD intracellular fate and clearance profile are still not fully exploited. The present study mainly focused on the various effects along with its clearance and biodistribution in different parts of the body.

Our results suggest that QDs did not affect the feed and water intake of animals. These results are in accordance with our previous study in iron oxide nanoparticles (IONPs) where administration of nanomaterial did not induce any significant changes in the eating or behavioral changes in the treated animals [36].

Clearance profile suggested that QDs were able to excrete out from the body of the animals efficiently after the administration in a few days only. Although the variations were also observed based on the individual animals, excretion analysis suggested that QD nanomaterials were excreted out of the body of the animals through urine and feces. The elimination process seems to be connected with the distribution of nanomaterials in different organs of the body. Our results are in accordance with previous studies which indicated a higher concentration of NPs in faces [36, 37]. Nonetheless, the hepatobiliary process is a possible reason behind the higher excretion from the liver to the intestine through fecal matter rather than urine [37]. The elimination of nanoparticles through feces was more than in the kidney, and it may be also confirmed by the deposition of QDs in the kidney in the present study. In contrast, QDs were found to eliminate through urine not from the feces [38].

Biodistribution of NPs to different organs results from systemic circulation, and NPs may get delivered to the same organs despite different administration routes. Through systemic circulation, NPs reach various organs such as the liver, kidney, spleen, testes, lungs, heart, and brain [39]. Bioaccumulation showed a somewhat linear dose dependence except for the brain which is in accordance with the report by Lasagna-Reeves who analyzed the bioaccumulation of gold NPs in rats [40]. In yet another study on gold NPs, the spleen was suggested to be the key organ in NP metabolism [29]. Moreover, delivery of NPs to the spleen is mainly due to action of the immune system via macrophages [41]. However, wide variation in dose, particle size, and experimental designs makes it difficult to outline a generalized summary of the bioaccumulation of particles. But most of the studies so far report maximum accumulation in the spleen and liver, suggesting hepatobiliary mode of clearance [29, 30]. In addition, the liver is a detoxifying organ, and due to the presence of Kuffer cells (resident macrophages), NPs may get deposited in the liver. Similarly, a previous study also reported deposition of NPs in red and white pulp zone of the spleen [41]. Moreover, the liver and spleen have been observed as major deposition sites for intravenously injected metallic NPs [42]. The present study is consistent with previous studies with metallic nanomaterial where deposition of nanoparticles was found to be high in the liver and spleen as compared to other organs after intravenous administration [36, 42]. However, the size of NPs also accounts for the deposition of NMS. NPs were also deposited in the kidney in a significant amount in the present study. Elimination of NPs mainly occurs in the kidney with urine from the blood circulation that indicates the deposition of NPs in the kidney which ultimately results to various adverse effects such as biochemical alterations and morphological changes in the kidney.

The administration of NPs leads to their circulation in the blood where interaction with immune cells and plasma protein may occur. The interaction may result in the progression of oxidative stress, reduced level of antioxidant system, increased number of immunological cells, and reduced numbers of blood cells. The present study suggests that the inflammatory response is generated through induction of immunological changes, i.e., alteration in blood cell counts after the administration of different doses of QDs. The administration of nanomaterials leads to the circulation in the blood where NPs interact with blood cells and plasma protein. These interactions may result in several possible pathways to begin such as hemolysis and complement activation. The report suggests that different QDs have been observed to show hemolytic activity in RBCs [43]. It has been previously reported that the free radicals generated by NPs are responsible for hematological changes [44, 45]. When QDs interact with constituents of the blood, it results in the release of free Cd^2+^ ions that in turn lead to immunogenic and hematological changes. This affects WBC, platelets, and hemoglobin and interferes with the maturation of RBC [46]. Thus, the results indicate that the administration of varying doses of CdSe can induce an inflammatory response and bring about considerable changes in hematological factors which are in concordance with the studies of Rezaei [47]. They postulated that titania NPs were capable of inducing significant changes in blood cells and also observed an increase in WBC count.

The liver and kidney are considered to be the first-line organs to protect them from any xenobiotic components. In the present study, therefore, we measured the liver and kidney profiles. The results from the biochemical panel of assays suggested that the QDs induce the generation of liver and kidney biomarker metabolites implying QD-induced hepatic and nephrotoxic damage at both acute and subchronic levels [48]. Thus, as discussed above, we observed elevated levels of ALP and AST levels in the CdSe-treated QDs which can be associated with liver dysfunction, diseases of the biliary system, and pancreatic damage. The administration of QDs at a concentration above 20 mg/kg induces hepatocellular damage, thus confirming hepatic injury to the treated rats.

NPs may involve in ROS induction and generation of oxidative stress which eventually results in alteration in the antioxidant system [36, 49]. Metallic NPs have been reported to induce oxidative stress and, thus, interfere with the antioxidant system of animals [50]. The present study revealed that QDs treated showed a significant increase in oxidative stress as compared to the control one. A compromised and altered antioxidant system has the need to be observed in the QDs-treated groups. Moreover, a dose-dependent increase in oxidative stress was observed after the treatment of the ZnO QDs [51]. Thus, it can be inferred from the results that the accumulated CdSe particles induced oxidative stress, which in turn was responsible for the kidney and DNA damage as well as the dysfunction of the antioxidative system in the brain.

## 4. Materials and Methods

CdSe quantum dots were synthesized from the precursors CdO and elemental Se using a protocol with slight modifications [52, 53]. In brief, Se precursor stock solution was prepared by mixing 30 mg of Se to 5 mL of 1-octadecene in a 10 mL flask which was clamped on a hot plate, to which 0.4 mL of trioctylphosphine (TOP) was added. The solution was heated to 40°C and stirred for about 15 min for complete dissolution of Se powder. This Se stock solution was stored at room temperature (20°C) in air tight bottle. Cd precursor was prepared by adding 13 mg of CdO to a 25 mL flask containing 0.6 mL of oleic acid and 10 mL of octadecene, which was then heated gradually to a temperature of 225°C till the solution turned colourless. A 1 mL of the previously prepared Se solution was added to the hot cadmium solution dropwise. The physical size and morphology of the products depend on the reaction time and temperature. Further details are available in the previously conducted study [28].

### 4.1. Animal Treatment

Male Wistar rats (8–10-week-old with body weight of approx. 230 g) were used for CdSe QD toxicity study. The rats were obtained from the Central laboratory for animal resource (CLAR) Animal House, JNU, New Delhi, India for the study. The animals were kept in the animal house under stress-free, controlled temperature (21 ± 3°C), hygienic atmosphere at 12 hours day/night cycle and were supplied with food pellet and water. The animals were divided randomly into four groups of six animals in each. CdSe QDs were intravenously injected in the caudal vein and kept for 30 days. The QDs were used at doses of 10, 20, 40, and 80 mg/kg of particles. One group was injected with phosphate buffer saline (PBS) and was considered the control. However, other four groups were treated with the different doses of QDs, i.e., 10, 20, 40, and 80 mg/kg NPs. All the experiments were performed as per the guidelines and approval of Animal Ethics Committee of JNU, New Delhi, India.

#### 4.1.1. Sample Collection

Sampling was done by withdrawing 1 mL of blood from retroorbital sinus via a heparin-coated capillary and was collected in a tube containing 20 mg/mL EDTA to prevent coagulation. Prior to sampling, animals were anesthetized using 0.3 mL/250 mg ketamine/xylazine. Sampling was done at weekly interval from both the control and treated groups of animals. The collected blood sample was subjected to centrifugation at 2000 g for 15 min. The serum was collected and stored at −20°C, and the pellet was used for hemolysate preparation.

#### 4.1.2. Hematological Assay

The pellet obtained was washed thrice with PBS buffer, centrifuged at 2000 g for 15 min at 4°C, and thereafter mixed with ice cold distill water (1.9 mL) and packed cell volume suspension (0.1 mL). The hemolysates was stored at −20°C for further analysis. Automated hematological analyzer (KX-21, Sysmex, Transasia, India) was used for various hematological analysis such as red blood cells (RBC), hemoglobin concentration (HGB), hematocrit, white blood cells (WBC), platelets count (PC), mean corpuscular volume (MCV), and for the determination of percentages of lymphocytes and monocytes.

#### 4.1.3. Biochemical and Oxidative Stress Measurement

Several biochemical assays related to the liver, kidney, and brain functions was conducted on Biochemical Autoanalyzer (Type 7170, Hitachi, Japan). Different liver and kidney marker tests were also performed. Antioxidant stress markers such as superoxide dismutase (SOD), catalase (CAT), glutathione peroxidase (GPx), and lipid peroxidase (LPx) were analyzed using respective enzyme-based kit method.

#### 4.1.4. Determination of Body Weight and Coefficients of Organs

The animals (control and treated) were scarified by overdose of anesthetic ether and cervical dislocation. Thereafter, body weight of all groups of animals was recorded. Organs (the spleen, liver, lungs, kidney, brain, and testis) were removed, and weight was recorded immediately. Coefficient of organs was then calculated by taking the ratio of organ wet weight (mg) to body weight (g). Experiments were performed in specifically designed metabolic cages in order to collect the urine and feces of the Wistar rats. Cd concentration was estimated in the collected urine and feces so as to determine any changes in the metabolic activity after treatment with the QDs.

#### 4.1.5. Determination of Cadmium Concentration

Quantitative measurement of Cd was performed by removing and burning the organs at 200°C for around 20 minutes. Atomic absorption spectroscopy (AAS) analysis was performed using 1 g of the organ power. AAS analysis was performed after complete digestion of the samples and readings were taken against standard Cd stock solutions. Cd concentration in the spleen, liver, kidney, testis, brain, heart, and lungs was determined to understand the bioaccumulation rate. Metabolic rate of the treated and control groups of Wistar rats was recorded at regular time intervals to monitor any variations brought about in the normal metabolic activity after administration of the QDs.

### 4.2. Statistical Analysis

Statistical analysis was performed via one-way analysis of variance (ANOVA) followed by 2-sample, *t*-test comparing the control and treated groups. The significance level of 0.05 (95%) was ascribed. Results are presented as mean and standard deviation of six independent replicates.

## 5. Conclusion

The administration of CdSe QDs in Wistar rats did not induce major changes in the food and water intake. The spleen showed the greatest accumulation of CdSe particles, whereas the testis showed the least accumulation. In addition, Wistar rats treated with high concentrations of QDs experienced changes in their hematological and biochemical indices. Additionally, as QDs degraded over time, Cd2+ ions were released, which in turn caused immunogenic changes that damaged the kidneys and liver. The generated Cd^2+^ ions induced alteration in red blood cell number, bringing about decrease in cellular antioxidants, thereby enhancing the oxidative stress. In conclusion, the results of this study suggest that high doses of CdSe QDs may be toxic. However, the QD concentration used for the analysis of bioimaging is typically lower than the concentration we used for the evaluation of toxicity. Consequently, the effectiveness of CdSe QDs in bioimaging applications should not be completely disregarded.

## Data Availability

All data related to this article is available on request to first and corresponding author.
